# Surgical Clinic of Prof. Westmoreland

**Published:** 1873-03

**Authors:** C. M. Hill

**Affiliations:** Atlanta


### SURGICAL CLINIC ATLANTA MEDICAL COLLEGE—SERVICE OF PROF. W. F. WESTMORELAND.

REPORTED BY C. M. HILL, M.D., ATLANTA.

T. G., colored, aged thirty-seven; baker; well developed, but for some time past rather emaciated. Has suffered several months with an abscess over the right clavicle, which still discharges a slight quantity of pus. Between the clavicle and nipple of same side there is a considerable tumefaction, apparently the result of a deposit of some kind under the pectoral muscles. To the right of the nipple, and in the vicinity of the axilla, there is an extensive ulcerative service with one or more deep openings, which discharges upon pressure a small quantity of pus. A probe introduced into these openings came in contact with denuded bone, evidently the third or fourth rib. The patient stated that six years ago he contracted syphilis. The primary development was followed by the usual train of constitutional symptoms. Anti-syphilitic remedies were administered, and the symptoms rapidly disappeared, to re-open again in eight or ten months; since which time there has been only a few months interval at any time when the patient was exempt from some of the phenomena of this terrible scourge. For seven months he has suffered great pain over the clavicle, and, more recently, under the pectoral muscles, which have been followed by the above mentioned ulcerated and suppurative points in these localities.

From the history and symptoms above given, Dr. Westmoreland was confident, notwithstanding the reverse opinion of those who had previously treated the abcesses, that the whole trouble was specific, and that we had in this case a well-marked tertiary syphilis; that the abcess over the clavicle was evidently the result of syphilitic periostitis, and that under the pectoral muscles was the same disease of the third or fourth rib, or both.

An opening was now made under the inferior border of the pectoralis major muscles of sufficient size to drain well the discharge from the necrosed ribs, and a large tent introduced to insure the drainage. Iodine of potassium was given in large doses, as the following formula will show: ℞.—Iodide of potassium, ℥ ij; syrup stillingia, ℥ ij; syrup gentian, ℥ vj. Of this mixture the patient was ordered to take half an ounce three times a day, making one hundred and sixty grains of the iodide of potassium each day. Professor Westmoreland impressed upon the patient the importance of taking the medicine largely diluted with water; each dose should be taken in a full glass of water, and if it induced any burning or unpleasant sensations in the stomach, to still further dilute it. He stated that the only trouble he had found in the administration of heroic doses of the iodide of potassium in tertiary syphilis was, in some cases, the unpleasant effect on the stomach, which can always be prevented by diluting it witha large quantity of water; and if this precaution be taken, the dose may be increased to even three hundred grains a day without unpleasant results, as he had often demonstrated.

This plan of treatment was commenced on the third of November last, and in three or four days the improvement was marked. The effect of the remedy was magical in removing the periosteal pain which had so annoyed the patient. With an occasional interval of a day or two, this treatment was continued for one month with the most marked improvement; so marked that the patient, thinking himself well, disappeared from the clinic until the 1st February, when he returned with the ulcer again discharging. The same remedy was again administered, with the same happy results.

Dr. Westmoreland stated that this case illustrates what the physician has to contend with in the treatment of this disease, particularly in the ignorant classes. That it is difficult to impress upon them the importance of continuing treatment a sufficient length of time to eradicate the disease; that when all the symptoms have disappeared, as in the above case, still, the germ is not destroyed, and will, with great certainty, show itself again in some form. Often he has been annoyed, even by an intelligent class of patients, who become restive under restraint and medication just so soon as the last external symptoms disappear, and often refuse positively, or at best take the remedies irregularly, and at the end a few months return with the appearance of the disease and a criticism upon your mode of treatment. Sometimes, however, the physician is at fault, when, either through ignorance of the disease, or cupidity, he becomes desirous of terminating the case, he dismisses his patient as cured just so soon as the last external symptom disappears. He attempted to impress the class with the importance of watching their cases for months, or even years, after all symptoms have disappeared.

CASE II.—M. C., colored, aged fifteen years; servant. This boy has for two or three weeks suffered from an ordinary gonorrhœa. From some cause, had a day or two ago a suppression of the gonorrhœal discharge, and we find him suffering from an acute pain in the right testicle, extending in the direction of the spermatic cord, with considerable tumefaction. He has, also, some pain and swelling in the knee. Dr. Westmoreland regarded this case as a type of a case of metastasis of the specific inflammation to the testicle and knee-joint. This metastasis or transfer of the inflammation from the urethra to the testicle is not unusual, and sometimes is the result of treatment, and then, in his opinion, is not metastasis proper but a continuation of the inflammation by continuity of surface, as where, from an irritating injection thrown up the urethra as far as the seminal ducts, the inflammation then extends itself to the epididymis, making epididymitis, and not orchitis proper.

In the absence of any history in this case certainly, it is impossible to speak positively in regard to this little patient; but from the slight inflammation which exists in the knee, he was impressed with the opinion that we have here a true metastasis. A saline cathartic was ordered, and an application of collodion was made to the scrotum, particularly on the side of the inflamed testicle, and in this way to give support and uniform compression of the inflamed organ. The collodion was ordered to be applied two or three times a day, or as often as there were breaks in the covering made by the collodion.

Professor Westmoreland was of the impression that the beneficial effect of this remedy was not dependent upon any direct action on the inflamed tissue, but purely mechanical in giving support and uniform compression. This application was kept up for three days, when the patient represented himself greatly relieved, disappeared from the clinic, thinking himself cured. About three weeks later, he again presented himself, and with the most intense suffering I have ever witnessed. Upon examination, the testicle was found but slightly swollen, but the most excruciating pain was produced by the slightest touch. He stated that he had been in this condition for twenty-four hours. The tumefaction was not one-third so great, nor did there appear to be so great an inflammation as in the attack three weeks before. Professor Westmoreland stated that the case was a very rare one; that instead of the effusion taking place in the tunica vaginalis testis, that the effusion was under the fibrous covering of the testicle—a membrane unyielding and non-elastic, consequently the excruciating pain from such a slight effusion. The pain is not so much the result of the intensity of the inflammation as the compression of the testicle from the effusion under its fibrous covering. If we are correct in our diagnosis, we can in a very few minutes relieve, almost entirely, the sufferings of this patient; if we are mistaken, perhaps we will add to his sufferings. If we are correct, his acute suffering is dependent upon the compression of the testicle—the result of the effusion of serum under the fibrous covering of the testicle. If we remove this fluid, we remove the compression, and his sufferings will cease. If we are mistaken in diagnosis, the punctures to remove an imaginary fluid will wound the testicle and necessarily add to his suffering. Professor Westmoreland then, with a small and narrow bistoury, made two or three punctures over the testicle, and a clear liquid passed out in a jet through the openings thus made. The relief was magical. His sufferings almost entirely subsided, and without further treatment, other than a support to the testicle, quiet and proper diet, the patient had a good recovery.

